# Medial pivot total knee arthroplasty

**DOI:** 10.1016/j.jcot.2025.103220

**Published:** 2025-10-01

**Authors:** Ahab G. Alnemri, Ryan Turlip, Amanda Moser, Ashleigh Bush, Jake Farrar, Neil P. Sheth

**Affiliations:** Department of Orthopaedic Surgery, University of Pennsylvania, Philadelphia, PA, USA

## Abstract

Over recent years, medial pivot total knee arthroplasty (MP-TKA) has gained traction as it more closely replicates native knee kinematics. This review traces the evolution from early TKA designs through modern “ball-and-socket” concepts and describes how MP implants stabilize the medial compartment while allowing for physiologic lateral rollback. We aim to provide an overview of the biomechanical rationale of the MP-TKA design and analyze contemporary literature on survivorship and patient satisfaction.

Recent fluoroscopic and motion capture investigations confirm that MP devices can reduce paradoxical anterior translation and improve sagittal-plane stability. However, pivot behavior varies by activity, implant geometry, and alignment strategy, and differences in kinematic measurement methods, such as low-point versus contact-point tracking, can influence interpretation. Despite these methodological differences, many MP designs demonstrate favorable in vivo performance.

Several small-to-large clinical series, registries, and randomized trials report promising patient-reported outcome measures and survivorship rates comparable to, or better than, traditional posterior-stabilized TKA. However, comparative studies have noted higher revision rates with specific MP implants. Design variations, surgical technique, and patient selection may significantly affect outcomes.

While preliminary findings are encouraging, large-scale and long-term research is needed to clarify whether enhanced kinematics consistently translate into clinical benefit. This review highlights key implant designs, biomechanical considerations, and evolving evidence to guide surgeons who are considering MP-TKA.

## 1 Introduction

Total knee arthroplasty (TKA) is a successful treatment for end-stage arthritis, providing pain relief and functional restoration. However, despite advances in technique, perioperative care, and implant design, 10 %–15 % of patients remain dissatisfied.^1^ Enhancing patient satisfaction following TKA remains a challenge, and one area of particular interest is the replication of native knee kinematics. Early TKA designs exhibited abnormal or non-physiologic knee motion, prompting the development of the medial pivot (MP) or “medial constrained” bearing design. This review will cover native knee kinematics, previous TKA implant designs, kinematic implications of the MP design, and clinical outcomes following MP-TKA.

## 2 Methods

A systematic literature search was performed to identify studies evaluating the biomechanics, kinematics, clinical outcomes, and survivorship of MP-TKA. PubMed/MEDLINE, Embase, and Scopus were queried for publications from January 2000 through April 2025. Search terms included combinations of "medial pivot," "total knee arthroplasty," "knee replacement," "kinematics," "fluoroscopy," "survivorship," "patient satisfaction," and "clinical outcomes." Inclusion criteria were studies reporting original data on MP-TKA biomechanics, fluoroscopy-based kinematic analyses, clinical outcomes compared with other TKA designs and within MP-TKA alternatives, patient satisfaction, or implant survivorship. Case reports, editorials, letters, and those focusing exclusively on surgical techniques or perioperative management without outcome or kinematic analysis were excluded. Abstracts and titles were initially screened, followed by a full-text assessment for eligibility. Additionally, the references of selected studies were manually reviewed to ensure inclusion of all relevant literature.

## 3 Native knee kinematics

The knee consists of two articulations - the patellofemoral and the tibiofemoral joint. The patellofemoral articulation serves as a connection between the extensor mechanism, allowing for the transmission of tensile forces from the quadriceps and acting as a lever arm to amplify to force produced by the extensor mechanism.^2^ The patellofemoral joint is a sliding articulation, with maximum bony contact occurring at 45° of flexion. The medial patellofemoral ligament and the medial retinaculum provide stability within the trochlear groove. The tibiofemoral articulation transmits body weight. Normal range of motion is 3° of hyperextension to 155° of flexion, with calf-thigh impingement limiting maximal flexion. Most activities of daily living necessitate a 110° arc of motion.^3^

Knee kinematics are driven by asymmetric articular surfaces where the medial tibial plateau is concave, and lateral is convex. Additionally, the medial meniscus is “C”-shaped, providing an anterior and posterior lip that resists translation. Conversely, the lateral meniscus has a circular shape allowing 15° of anterior to posterior motion and 11 mm of translation.^4^ From a ligamentous perspective, the anterior cruciate ligament prevents anterior subluxation of the tibia on the femur, and the posterior cruciate ligament prevents posterior subluxation. The lateral collateral ligament (LCL) provides stability particularly in varus stress and posterolateral rotation of the tibia. Conversely, the medial collateral ligament (MCL) offers stability to the knee during valgus and rotational stresses.

**Fig. 1:**
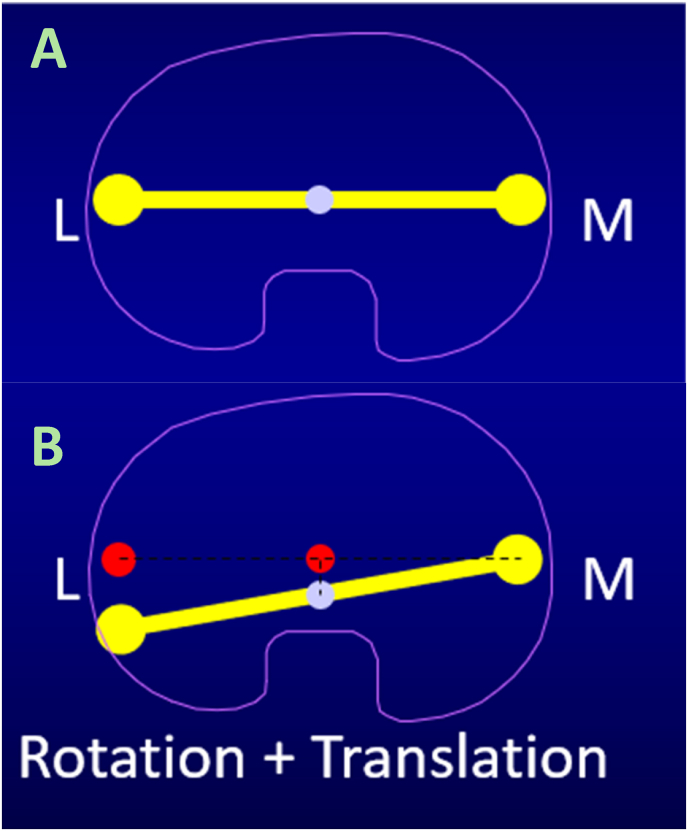
Patellofemoral Rollback of Native Knee. (A) Static knee. (B) Following 110° arc of typical flexion, the medial femoral condyle remains stationary (pivot point), while the lateral femoral condyle is translated posteriorly and the femur is internally rotated. L = lateral; M = medial.

The first major study to evaluate biomechanical differences between the femoral condyles utilized magnetic resonance imaging (MRI) to assess cadaveric knees. The study found that during flexion, the medial condyle has a static center of rotation without anteroposterior (AP) translation. Conversely, the lateral condyle translates posteriorly relative to the lateral tibial plateau, resulting in relative internal rotation of the tibia about a medial pivot.^5^ This phenomenon, known as femoral rollback, was first described by Giovanni Alfonso Borelli (1608–1679). He noted that as the knee goes into flexion, the lateral femoral condyle's center of rotation moves posteriorly, more so than the medial condyle (Fig. 1). This pattern is critical for allowing maximal knee flexion and avoiding tibiofemoral impingement.^6^

During walking, the lateral knee acts as the pivot point as gait begins at toe off. The femur is positioned anteriorly and is externally rotated on the tibia. Transitioning to heel strike in the gait cycle, the knee extends while the femur undergoes internal rotation and posterior translation. This phenomenon is known as the “screw-home” mechanism, characterized by external rotation of the tibia by 5° in the final 15° of flexion.^7^^,^^8^ Anatomical studies using MRI demonstrated the average of all helical axes of the knee was located in the medial condyle and approximated at the epicondylar axis.^9^

Daily activities primarily occur between 10° and 120° of knee flexion, constituting a 110° arc of motion. The articular surfaces of the femoral condyles are circular from a sagittal view, and the marked asymmetry between the two compartments drives knee kinematics. This asymmetry results in posterior translation of the lateral femoral condyle during flexion, accompanied by tibial internal rotation (or femoral external rotation) during the first ∼15° of flexion. These coupled motions are essential for providing terminal deep flexion, tibiofemoral stability, and characterize the medial pivoting of the native knee.^10^ Conventional TKA does not consider native knee kinematics, whereas MP designs aim to recreate them to potentially improve functional outcomes.

## 4 Previous TKA implant designs/history

Initial generations of TKA implants drew inspiration from the “4-bar link” theory.^11^ According to this theory, the center of rotation within the knee is the intersection of the cruciate ligaments. Consequently, as the knee flexes, the center of rotation shifts posteriorly, facilitating femoral rollback. First-generation TKAs from the 1980–1990s therefore featured femoral components with a J-curve to support femoral rollback. However, these implants experienced paradoxical anterior translation at approximately 65–70° of flexion due to tibiofemoral incongruity in flexion and loss of stabilization.^12^, ^13^, ^14^ This paradoxical motion also resulted in audible impingement of the patellofemoral and/or spine-cam interfaces that was linked to anterior knee pain.^15^ The resulting paradoxical motion was first scrutinized when researchers noted the radius of the lateral femoral condyle was slightly smaller than the radius of the medial femoral condyle, but the center of each was positioned on a single axis.^16^

Second-generation implants shifted focus to the concept of a single axis of knee rotation. Blaha et al. used cadaveric specimens to analyze the kinematics of the knee through flexion-extension and internal-external rotation, and created the concept of the quadriceps vector (Qv) using a 3D muscle model.^17^^,^^18^ The Qv was found to pass from the top of the patella to the anterior femoral neck and just lateral to the femoral head. The closest alignment of the Qv was with a line connecting the center of the femoral head to the center of the medial femoral condyle, referred to as the spherical axis.

This body of research demonstrated that the knee had a single center of rotation and drove the second generation of knee prosthetic design. The primary objective of these novel designs was achieving symmetric rollback through the implementation of a single radius of knee rotation; however, their limited tibiofemoral congruency often resulted in instability.^12^ Fluoroscopic analysis revealed second-generation posterior-stabilized (PS) TKA implants demonstrated physiological femoral rollback on the lateral knee but excessive motion in the medial knee due to this lack of congruity.^19^

## 5 Medial pivot TKA design & kinematics

The MP design aims to replicate key aspects of native knee kinematics that previous generations have failed to achieve. By utilizing a stable pivot medially and arcuate motion on the lateral condyle, this design maintains medial conformity and stability throughout the complete range of motion.^12^ The constant radius is crucial for maintaining consistent tension on the collateral ligaments, which allows early motion, restoration of patellar tracking, and a constant contact area throughout motion.^20^^,^^21^ The medial ball-in-socket design offers a highly conforming implant with a large contact area, resulting in low contact stress and minimal edge loading.^22^ In contrast, the lateral arcuate trough provides less conformity compared to the medial tibial plateau facilitating posterior translation of the lateral contact point during deep flexion and recreating native posterior femoral rollback.

**Fig. 2:**
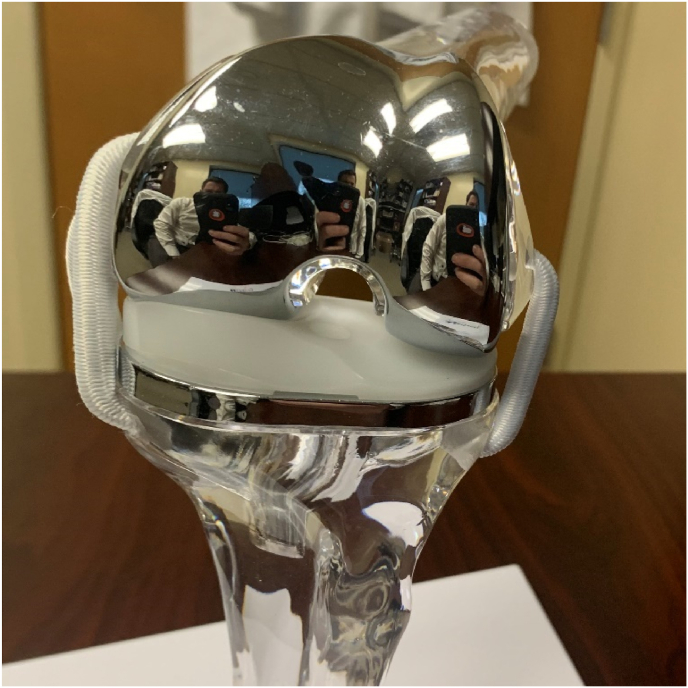
Medacta GMK sphere.

**Fig. 3:**
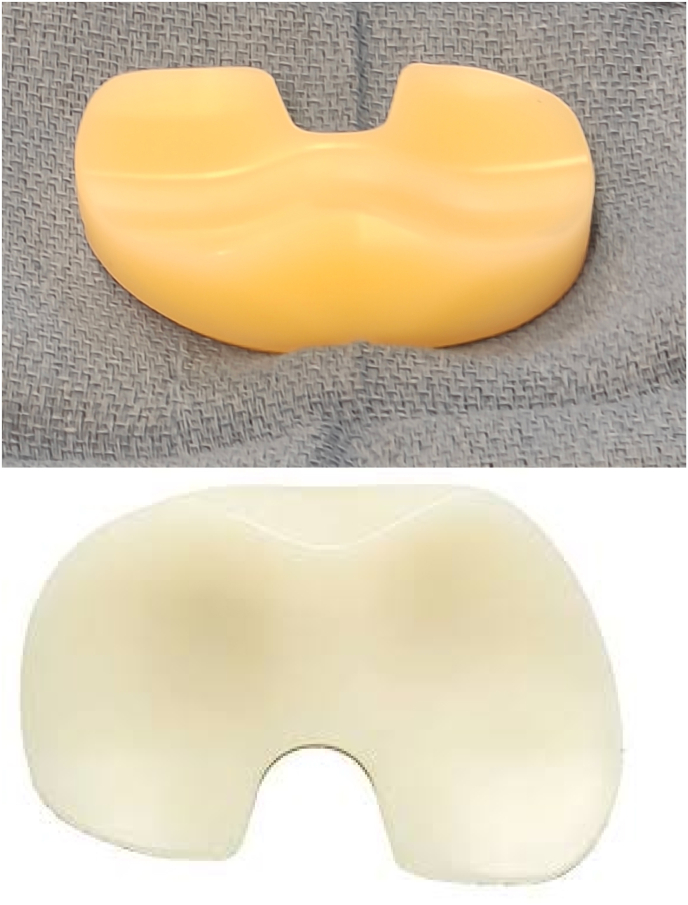
Asymmetric polyethylene insert.

**Fig. 4:**
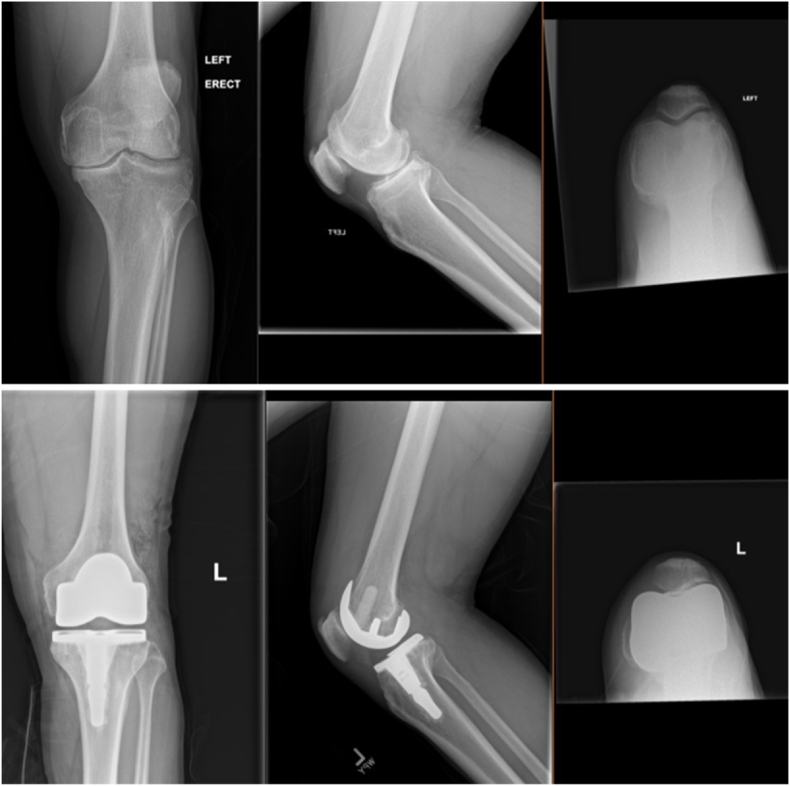
Pre- & post-operative radiographs demonstrating a medial pivot implant.

MP-TKA options include the Microport Advance and Evolution, Medacta GMK Sphere, MatOrtho MRK and SAIPH, Zimmer Biomet Persona Medial Congruent, and the Smith and Nephew Journey II (Fig. 2). Variations in design of the tibial component include an asymmetric baseplate versus an asymmetric polyethylene insert (Fig. 3). Variations of the femoral component include single- or dual-spherical condyles, symmetric versus asymmetric condyles, and a deepened versus lateralized groove.^23^ It is important to consider these alternative designs, as they likely render differences in kinematics which may impact clinical outcomes and implant survivorship. Many modern designs are a medial ball-and-socket design, or a pure MP design, which have a fully congruent medial articulation to avoid paradoxical anterior translation. The lateral compartment is a round femoral component on a flat or slightly convex articulation, which helps to replicate natural movement of the knee. Another design is a hybrid medial congruent system, such as the MC polyethylene (Zimmer Biomet, Warsaw, IN), which reproduces the medial pivot where the dwell point is posteriorly positioned. Overall, the design goal is to achieve a “natural” knee motion given the trend of performing TKAs in younger, more active patients. Fig. 4 depicts pre- and post-operative radiographs of an MP-TKA.

Methodological differences in how kinematics are measured and interpreted across studies can significantly influence outcomes and complicate direct comparisons of MP designs, implant performance, and clinical implications. Two commonly used conventions for assessing rotational motion are the Grood and Suntay system and Cardan angles. The Grood and Suntay system utilizes a floating axis approach to separate flexion-extension, abduction-adduction, and internal-external rotation, and is frequently applied in dual-plane fluoroscopy and computational modeling.^24^ Cardan angles describe joint motion using fixed axes and a defined sequence of rotations, often used in single-plane fluoroscopy studies. These differing conventions can yield distinct interpretations of joint motion depending on the rotation sequence applied.

In addition to rotational conventions, the International Society of Biomechanics (ISB) provides standardized recommendations for joint coordinate systems to help ensure consistency in anatomical landmark identification and axis assignment. These standards are useful when comparing studies that use different imaging modalities or modeling techniques. Furthermore, differing methods for quantifying condylar motion (e.g., low-point or contact-point kinematics) can also affect interpretation. Low-point kinematics track the lowest point on the femoral condyle relative to the tibial plateau and are sensitive to implant geometry, while contact-point kinematics estimate the centroid of contact between femoral and tibial components, and offer insight into load-bearing behavior. For example, Scott & Imam et al. employed low-point kinematics with single-plane fluoroscopy, whereas Yu et al. used contact-point analysis with dual-plane imaging. It is important to acknowledge these methodological differences when comparing outcomes across studies on MP designs.^25^^,^^26^

In 15 patients (16 knees) with Medacta GMK Sphere implants, Scott & Hellie et al. used single-plane sagittal pulsed fluoroscopy to assess dynamic flexion (step-up/down, pivoting) and static deep-flexion (kneeling, lunging). 3D shape-matching (accuracy <1° rotation, <1 mm translation) was used to produce Cardan-angles with AP condylar translations referenced to tibial landmarks.^27^ Across activities, the medial condyle translated minimally, while the lateral condyle rolled back proportionally to tibial internal rotation. During stair-stepping, tibial internal rotation averaged 6° with ∼6 mm lateral rollback, and pivoting produced 11° rotation about a stable medial axis. No paradoxical anterior translation was observed, supporting replication of native medial pivot mechanics.

Using dual-plane fluoroscopy and motion capture with the Grood & Suntay convention, Hamilton et al. evaluated 7 MP-TKA patients and 8 nonsurgical controls during chair rise, lunge, step-down, gait (with and without 90° turn), and seated knee extensions.^28^ In MP-TKAs, the primary pivot, defined by the projected central rotation axis, was central during chair rise, lunge, gait, and turning. Step-down showed a medial pivot, while extension shifted from central to lateral. The medial low-point showed minimal anterior translation (<1 mm) in lunge and gait, and ∼2.4 mm during extension, although total AP motion varied by task and participant. Compared to controls, MP-TKAs had reduced ROM in several degrees of freedom, especially at higher flexion, while controls showed greater physiologic variability.

Although MP-TKA designs aim to constrain rotation around a fixed medial axis, recent fluoroscopic studies show that pivot location varies by activity, implant design, and alignment strategy. As stated, Hamilton et al. found that pivot behavior shifted across tasks. Yu et al. reported a stable medial rotation center in MP implants versus lateral pivoting in PS designs during step-up (both studies defined pivot by the central rotation axis).^26^ Kaneda et al. used AP condylar translation to define pivot and showed increased medial pivot motion in kinematically aligned TKAs compared to mechanically aligned TKAs.^29^ These findings indicate that while the design intent is a consistent MP, actual kinematics are dynamic and affected by implant geometry, alignment, and patient-specific biomechanics. Future studies should report pivot location by activity and assess its association with clinical outcomes.

## 6 Patient satisfaction and implant survivorship of medial pivot TKA

While early reports on MP-TKA have shown promising results in terms of survivorship and patient satisfaction, these outcomes must be interpreted in the context of biomechanical performance and implant design. As stated, a meaningful analysis requires not only reporting outcome metrics but also understanding how these metrics vary across implant types, alignment strategies, and patient-specific factors. Clinical assessments such as the Knee Society Score (KSS), Forgotten Joint Score (FJS), Knee Injury and Osteoarthritis Outcome Score (KOOS), Oxford Knee Score (OKS), Western Ontario and McMaster Universities Osteoarthritis Index (WOMAC), EuroQol-5 dimension (EQ-5D), and other patient-reported outcome measures (PROMs) provide valuable insight into functional recovery and satisfaction.

Few studies directly compare these scores across MP and PS designs while controlling for surgical approach or native knee comparators (Table 1, Table 2). In a prospective randomized study of 600 patients, Obada et al. found that those who received MP implants scored significantly higher on the FJS (mean: 59.7) compared to those with PS implants (mean: 44.8), particularly in deep flexion and perceived joint stability.^30^ Additionally, the same study found that OKS and KSS were consistently higher in the MP group, while WOMAC for pain and stiffness were slightly better in the PS group at one and two years.

**Table 1:** Comparison of survivorship outcomes between studies.

Study, Year	Study Design	Implant Company	Sample Size (N)	Follow-Up Time (Years)	Comparator (Design)	Survivorship Outcomes
Alessio-Marzzola et al., 2022^32^	Systematic review	Various	3377 (34 studies)	7.1	Various	1.9 % revision rate at 10 years (MP)
Cacciola et al., 2021^33^	Systematic review	Various	2832 (18 studies)	8.1		97.6 % overall, 99.1 % free of infection, 99.6 % free of aseptic loosening. 2.4 % overall reoperation rate
Jenny et al., 2021^44^	Retrospective cohort	Various	577	13	CP	97 % (MP) vs 98 % (CP) survival at 13 years; 2 % overall revision rate
Øhrn et al., 2020^34^	Retrospective cohort	Various	6012 (Australia) + 298 (Norway)	9	CR (fixed-bearing)	Australia: 9-year survival: 94.8 % (MP) vs 96.4 % (CR); Norway: 9-year survival: 92.2 % (MP) vs 94.5 % (CR)
Cassar-Gheiti et al., 2020^36^	Systematic review and meta-analysis	Various	3684 (25 studies + registries)	6.9		Survivorship free of aseptic loosening ∼99 % at 6.9 years (25 studies); NJR: 15-year revision rate 4.6 %, AOANJRR: 15-year revision rate 8.1 %, LROI: 10-year revision rate 9.8 %
Bordini et al., 2016^43^	Retrospective cohort	MicroPort Orthopedics/Wright Medical	506	6.6	Various	10-year survival: 96.3 % (MP) vs 95.7 % (others); 3.1 % revision rate
Karachalios et al., 2016^38^	Retrospective cohort	MicroPort Orthopedics/Wright Medical	284 (225 patients)	13.4		Survival at 15 years: 97.3 % (all-cause), 98.8 % free of aseptic loosening

Abbreviations: MP, medial pivot; PS, posterior-stabilized; CR, cruciate-retaining; CP, central pivot; KM, Kaplan–Meier; NJR, National Joint Registry; AOANJRR, Australian Orthopaedic Association National Joint Replacement Registry; LROI, Dutch Arthroplasty Register; N, sample size.

**Table 2:** Improvement in (or Final Postoperative Scores) of Patient Reported outcome measures (PROMs) Across Studies.

Study, Year	Study Design	Implant	Sample Size (N)	Follow-Up Time (Years)	Clinical Knee Society Score (cKSS)	Functional Knee Society Score (fKSS)	2011 Knee Society Score	WOMAC	Oxford Knee Score (OKS)	KOOS	Visual Analog Scale (VAS)	Forgotten Joint Score (FJS)	EQ-5D
Obada et al., 2025^30^	RCT	MP	300	2	35.79	28.91		36.17	20.98			80 (postop)	
		PS	300	2	36.21	28.76		36.56	19.99			75.0 (postop)	
Kato et al., 2023^41^	Prospective Cohort Study	MP	70	1	9.8	24.2	12.6 (satisfaction). −2.5 (expectation)		13.5		39.9 (pain)	66.0 (postop)	
		PS	51	1	8.7	20.6	9.6 (satisfaction), −2.0 (expectation)		12.3		33.6 (pain)	58.3 (postop)	
Alessio-Marzzola et al., 2022^32^	Systematic review	MP	3377 (34 studies)	7.1	47.2	38.9		36.0 (8/18 studies); 40.2 (3/18 studies)	10.4	34.5		68.5 (postop, 2 studies)	
Cacciola et al., 2021^33^	Systematic review	MP	2832 (18 studies)	8.1	49.1	38.1		40.2 (3/18 studies) 36 (8/18 studies)	21.7 (5/18 studies)	39.3 (2/18 studies)		69.9 (postop, 2/18 studies)	
Jenny et al., 2021^44^	Retrospective case-control	MP	327	13	94 (postop)	76 (postop)			53 (postop)				
		CP	343		93 (postop)	77 (postop)			52 (postop)				
Batra et al., 2021^35^	RCT (bilateral TKAs)	MP	53	4			30.2 (satisfaction) 0.9 (expectation)		35.1				
		PS	53	4			27.4 (satisfaction), −0.4 (expectation)		34.7				
Chang et al., 2021^40^	RCT	MP	39	2	34.7	27.3		33.7	20.8	•			
		PS	40	2	35.6	29.3		36.1	19.9				
Kulshrestha et al., 2020^39^	RCT	MP	36	2			22.6 (satisfaction), 0.2 (expectation), 44.5 (objective), 43.7 (activity)					52.7	47.2
		PS	37	2			24.2 (satisfaction), 0.4 (expectation), 67.5 (objective), 42.9 (activity)					55.6	53.3
Karachalios et al., 2016^38^	Retrospective cohort	MP	284 (225 patients)	13.4	57.6	35.5		38.4	19.3				
Bae et al., 2016^42^	Retrospective cohort	MP	150 (125 patients)	5.2	30.5	32.3		18.5					
Hossain et al., 2011^37^	RCT	MP	40	2	33.3	26.8		28.9	15.4				
		PS	40	2	20.2	20.1		20.9	12.6				

Abbreviations: CP, central pivot; CR, cruciate-retaining; cKSS, clinical Knee Society Score; fKSS, functional Knee Society Score; KOOS-JR, Knee injury and Osteoarthritis Outcome Score for Joint Replacement; MP, medial pivot; N, sample size; SD, standard deviation; TKA, total knee arthroplasty; PS, posterior-stabilized; RCT, randomized controlled trial; VAS, Visual Analog Scale; WOMAC, Western Ontario and McMaster Universities Osteoarthritis Index; FJS, Forgotten Joint Score; EQ-5D, EuroQol 5-Dimension Health Questionnaire; OKS, Oxford Knee Score; ROM, Range of Motion.

A multicenter, double-blinded randomized controlled trial by De Groot et al. compared MP, PS, and rotating platform (RP) TKA designs in 210 patients.^31^ MP-TKA provided significantly better AP stability at early (30°) and mid-flexion (60°) compared to PS- and RP-TKA. However, despite these biomechanical advantages, improvements in the OKS, KOOS, and EQ-5D were comparable across all implant types. Notably, the study found that patients with ≥4 mm of AP laxity at early flexion, regardless of implant design, had significantly worse Kujala scores. These findings suggest that AP stability may correlate with specific domains of patient satisfaction, particularly those related to patellofemoral function, and highlight the complexity of linking biomechanical performance to clinical outcomes. Further research is needed to clarify how implant design interacts with patient-specific factors to influence satisfaction and function.

Alessio-Mazzola et al. conducted a systematic review of 34 studies encompassing 3377 MP-TKAs with an average follow-up of 7.1 years.^32^ The review reported a low revision rate of 1.9 % at 10 years and favorable clinical outcomes. Across studies, the mean postoperative range of motion was 117°, and KSS averaged 85.9 (clinical) and 84.7 (functional). OKS (mean 28.2), KOOS (mean 84.9), and FJS (mean 68.5) reflected high levels of patient satisfaction. Radiographic outcomes also showed reliable correction of deformities and stable implant positioning. While some studies reported complications such as stiffness (1.0 %) or infection (0.6 %), these were infrequent and not design-specific. The review concluded that MP implants provide excellent long-term survivorship and functional outcomes when appropriately selected and implanted, though it also noted the need for more high-quality, long-term comparative studies to clarify their role relative to other TKA designs.

Cacciola et al. performed a systematic review of 18 studies (2832 MP-TKAs) and reported a mid-term survivorship of 97.6 % at an average follow-up of 8.1 years.^33^ The most common reasons for reoperation were periprosthetic joint infection (0.9 %) and aseptic loosening (0.4 %). Across studies, the mean clinical Knee Society Score (cKSS) improved from 40.1 preoperatively to 89.2 postoperatively, and the functional score (fKSS) rose from 44.8 to 82.9. Range of motion increased by an average of 10.8°, along with improvements in WOMAC, OKS, SF-12, KOOS, and FJS. Registry data within Cacciola et al. included the Australian Orthopaedic Association National Joint Replacement Registry (AOANJRR), which demonstrated 94.5 % survivorship at 9 years for MP-TKA, compared with 96.4 % for CR and 95.9 % for PS. In the UK National Joint Registry (NJR), the 15-year cumulative revision rates were 4.6 % for MP, 4.3 % for CR, and 5.3 % for PS. A stratified analysis suggested that the Advance MP implant accounted for much of the excess mid-term revision risk (5.4 % failure rate) seen in the Australian registry, whereas other MP designs performed more comparably to CR and PS implants.

While the majority of the included studies in Cacciola et al. focused on the Advance MP design, the review also incorporated early data on second-generation systems such as SAIPH and K-Mod. These MP designs demonstrated promising outcomes (98.1 % survivorship), but were limited by the short follow-up duration (5–8 years). Cacciola et al.’s findings suggest that MP systems can offer reliable survivorship and similar improvements in PROMs compared to previous TKA designs, though further long-term comparative studies are needed to clarify design-specific performance and durability.

Øhrn et al. analyzed 6310 MP-TKAs from Australian and Norwegian registries, comparing five implant designs to over 70,000 cruciate-retaining (CR) TKAs.^34^ In Australia, MP implants had a higher revision risk (HR 1.4), with the Advance II MP (HR 1.7) and GMK Sphere (HR 2.0) showing the greatest risk. The most common reasons for revision in Australia were infection (27 %), pain alone (19 %), patellar erosion (13 %), and instability (9 %). In Norway, MP implants were most often revised for loosening or lysis (28 %) and instability (28 %), though the sample size was smaller (n = 298). Revision indications varied by implant and region, demonstrating that survivorship differs across MP designs and is influenced by implant geometry and surgical factors. Future research should aim to further stratify patient satisfaction and survivorship data by implant design, alignment technique, and biomechanical performance to better understand the clinical relevance of MP kinematics.

## 7 Conclusions

Medial pivot total knee arthroplasty is a significant evolution in implant design and aims to replicate native knee kinematics through a stable medial axis and controlled lateral rollback. Fluoroscopy and motion capture studies show that while these implants often meet their design goals, pivot behavior varies by activity, alignment strategy, and implant geometry. These factors must be considered when interpreting outcomes. Clinical data from registries, randomized trials, and long-term cohort studies suggest medial pivot designs offer comparable or superior survivorship and satisfaction, especially in joint stability and deep flexion. However, some designs show increased revision risk, underscoring the importance of implant selection and surgical technique. Further long-term studies are needed to clarify outcomes across designs, and surgeons should consider patient-specific factors, alignment, and implant geometry when selecting medial pivot systems.

## Statement on protected health information

There is no protected health information referenced in this review.

## Ethical statement

N/A.

## Statement on protected health information

The participants and any identifiable individuals consented to publications of their image.

## Credit author statement

Ahab G. Alnemri: Writing – Review & Editing; Ryan Turlip: Writing – Review & Editing; Amanda Moser: Writing – Review & Editing; Ashleigh Bush: Methodology, Investigation, Data Curation, Writing – Original Draft, Writing – Review & Editing, Visualization; Jake Farrar: Investigation, Data Curation, Writing – Original Draft, Visualization; Neil Sheth: Conceptualization, Methodology, Writing – Review & Editing, Visualization, Supervision, Project administration.

## Funding

This research did not receive any specific grant from funding agencies in the public, commercial or not-for-profit sectors.

## Declaration of competing interest

The authors declare the following financial interests/personal relationships which may be considered as potential competing interestsNeil Sheth reports a relationship with Medacta USA INC that includes: consulting or advisory and royalties. Neil Neil Sheth reports a relationship with Smith and Nephew Inc that includes: consulting or advisory. Neil Sheth reports a relationship with Zimmer Biomet that includes: consulting or advisory. Neil Sheth reports a relationship with ELSEVIER INC that includes: royalties.
